# Potential Donors' Awareness and Perceived Feasibility of Donating Human Milk in Australia

**DOI:** 10.1111/mcn.70224

**Published:** 2026-07-01

**Authors:** Melissa K. Hyde, Claire Newman, Abigail R‐A. Edwards, Vanessa Clifford, Barbara M. Masser, Laura D. Klein

**Affiliations:** ^1^ School of Psychology The University of Queensland Brisbane Queensland Australia; ^2^ Strategic Transformation & Readiness Australian Red Cross Lifeblood Melbourne Victoria Australia; ^3^ Strategy and Growth Australian Red Cross Lifeblood Alexandria New South Wales Australia; ^4^ Murdoch Children's Research Institute Parkville Victoria Australia; ^5^ Department of Paediatrics University of Melbourne Parkville Victoria Australia; ^6^ Department of Public Health and Primary Care National Institute for Health and Care Research Blood and Transplant Research Unit in Donor Health and Behaviour University of Cambridge Cambridge England UK

**Keywords:** attitudes, awareness, breastfeeding, human milk, milk banks, potential donors

## Abstract

Human milk banks rely on voluntary donations from lactating individuals producing more milk than their baby needs, to make pasteurised donor human milk (PDHM) for preterm babies. Demand for PDHM is increasing, as is the need for donors. Few Australian studies have explored potential donors' awareness and perceived feasibility of donating to a milk bank. We aimed to survey women breastfeeding their own child (< 24 months) or pregnant (and intending to breastfeed), and residing in major metropolitan areas of Australia, to determine their awareness of milk banks, and interest in and perceived feasibility (ease, eligibility) of donating to a milk bank. 1221 participants completed the survey (112 pregnant women, 1109 breastfeeding women). Most (78%) had heard of milk banks, and 81.7% lived in an area eligible to donate to a milk bank, yet only 11% were aware of a milk bank in their area. Most (60%) pregnant women would prefer to use online sources or health professionals to find information about donation. Most breastfeeding women (77%) were interested in donating milk, however only 26% thought it would be easy to meet milk bank requirements, and that they would be eligible to donate. Barriers to donating for breastfeeding women involved having no excess milk to donate, lack of time to express/donate, and lack of space to store milk. Awareness raising of local milk banks, promotion of donation information amongst healthcare professionals, and modifications to improve feasibility of donation will likely engage new potential donors and ensure sustainability of milk banks.

## Summary

- Most breastfeeding and pregnant women in Australia had heard of milk banks, and although most lived in an area where they would be eligible to donate, few were aware of a milk bank in their area.
- There was high interest among breastfeeding women about donating milk. However, only 26% thought donating would be easy, and they would be eligible to donate.
- Barriers for breastfeeding women who were interested in donating related to not having enough milk for their own baby, and/or not having enough excess milk to donate. Lack of time to express/donate and lack of space to store milk were also key barriers.

## 1 Introduction

When supply of mother's own milk is limited, pasteurised donor human milk (PDHM) is the preferred alternative source of nutrition for premature, low birth weight, or vulnerable infants (Moro et al. 2015; Quigley et al. 2024). Human milk banks are responsible for the supply of PDHM, with these milk banks reliant on voluntary donations from lactating individuals who are producing more milk than their baby needs. Following the longstanding national network of milk banking in Brazil (de Souza Rechia et al. 2016), PDHM use has increased in Australia, the US, and Europe, with steady growth in the volume of milk distributed and the opening of new milk banks (HMBANA 2025; Kearney 2023; Weaver et al. 2019). A survey of 108 UK neonatal units found that 34.9% expected demand for PDHM to increase (Shenker et al. 2023). As demand for PDHM grows, so too does the need for donors.

Most research focuses on recent donors to milk banks (Gutierrez dos Santos and Perrin 2022; Hyde et al. 2024; Kundisova et al. 2019; Li et al. 2024). Systematic reviews show that donors are motivated by a desire to help others, having an excess milk supply and wanting to avoid waste, support from health professionals, and awareness of the value/benefits of human milk (Doshmangir et al. 2019; Gutierrez dos Santos and Perrin 2022; Kundisova et al. 2019; Li et al. 2024). Common barriers to donating include lack of knowledge or awareness about milk banking and the donation process, lack of time due to the process (e.g., expressing) or return to work, religious beliefs, and logistical concerns (e.g., distance to milk bank) (Doshmangir et al. 2019; Gutierrez dos Santos and Perrin 2022; Li et al. 2024).

While information about donors is important, perhaps more critical is understanding interest amongst lactating women who are not current donors. Breastfeeding rates are often tracked as part of public health initiatives, however this information by itself does not accurately indicate how many in a population could potentially donate milk. Milk donors must be producing and storing more milk than their baby needs and meet health and lifestyle eligibility criteria for donation (Clifford et al. 2020). Moreover, potential donors must want to donate and be able to overcome individual or lifestyle barriers to donation.

Of eight studies focused on potential donors where a milk bank (non‐profit, hospital‐based) existed at the time of the study, and interest in donating to a milk bank was assessed, willingness to donate ranged from 11.0% to 87.5% (*Mean* 54.95%, *Median* 52.70%) (Bhoola and Biggs 2021; Ellsworth et al. 2021; Flores‐Rojas et al. 2024; Jackson et al. 2025; Jayakrishna et al. 2024; Nguyen et al. 2025; Tian et al. 2021; Virano et al. 2017; Zhang et al. 2020). Potential donor motives align with those of actual donors, being predominantly helping others/altruism, and having an oversupply and avoiding waste (Ellsworth et al. 2021; Hamilton and Middlestadt 2024; Jayakrishna et al. 2024; Mackenzie et al. 2013; Mampane and Wolvaardt 2024; Smyk et al. 2021; Castillo Valenzuela et al. 2024; Virano et al. 2017; Zhang et al. 2020).

As with recent milk donors, barriers to donation for potential donors centre primarily on lack of knowledge about milk banking generally and the donation process specifically (Bhoola and Biggs 2021; Biggs 2021; Ellsworth et al. 2021; Jackson et al. 2025; Mackenzie et al. 2013; Mampane and Wolvaardt 2024; Ouyang et al. 2025; Smyk et al. 2021; Tian et al. 2021; Virano et al. 2017; Zhang et al. 2020). Potential donors also cite barriers including concerns about having insufficient milk for their own infant or not having an excess supply to donate, the logistics of donating, time and effort involved, and lack of support from their partner or extended family (Bhoola and Biggs 2021; Biggs 2021; Brown et al. 2025; Hamilton and Middlestadt 2024; Jackson et al. 2025; Jayakrishna et al. 2024; Mackenzie et al. 2013; Mondkar et al. 2018; Smyk et al. 2021; Tian et al. 2021; Castillo Valenzuela et al. 2024; Virano et al. 2017).

To date, only three Australian studies have considered potential donors to human milk banks. One focused on deferral from donation (Hyde et al. 2023), one examined knowledge of and attitudes towards milk banking when no local milk bank was operational (Mackenzie et al. 2013), and the other was a descriptive qualitative study that interviewed 15 potential donors about their perceptions of donating to a milk bank (Newman et al. 2025). None estimated how many mothers would consider donating. Thus, the aims of this study were to survey women who are breastfeeding, or are pregnant with their first child and intend to breastfeed, about their:

1. Awareness of milk banks.
2. Preferred sources of information about milk donation (pregnant women only).
3. Level of interest and perceived feasibility of milk donation (breastfeeding women only).

## 2 Methods

### 2.1 Participants

Participation was open to Australian women who were breastfeeding/expressing milk for a child under 24 months of age and not currently donating to a milk bank, or pregnant with their first child and intending to breastfeed. Participants were eligible for the survey if they were aged over 18 years, could complete the survey in English, and gave informed consent.

### 2.2 Measures

Participants completed an online survey hosted on Australian Red Cross Lifeblood's (Lifeblood) REDCap platform. Survey topics and response options were informed by qualitative study findings with potential donors (Newman et al. 2025). The draft survey was piloted internally with researchers and milk bank operations staff to confirm clarity, appropriateness of wording, and alignment with study objectives. The survey (Appendix 1) consisted of a series of tick box, Likert style, and/or free‐text questions including:

- Demographics: e.g., age, location, blood/plasma donation history.
- Awareness of milk banks: previously heard of a milk bank or peer milk donation/milk sharing: tick box (yes, no, unsure); awareness of local milk bank: tick box (yes, no, unsure); name of local milk bank: free‐text responses; previous donation to a milk bank (yes, no).
- Preferred sources of information about milk donation (pregnant women only; based on prior work [Newman et al. 2025] in which women indicated they wanted to know about milk donation earlier): 5 sources (e.g., health care professionals, social media) with tick box (not at all, maybe, definitely), plus an ‘other’ free‐text option.
- Interest in donating milk (breastfeeding women only): tick box (not interested, unsure, interested a little, interested a lot); and if not interested, reasons why: 7 reasons (e.g., I don't have extra milk/more milk than my baby needs) with tick box (yes, no), plus an ‘other’ response with free‐text option.
- Ease of donating milk (breastfeeding women interested in donating only): tick box (very difficult, difficult, easy, very easy); and if difficult to donate, reasons why: 8 reasons (e.g., I don't have space to store extra milk for donation) with tick box (yes, no), plus an ‘other’ response with free‐text option.
- Perceived eligibility to donate milk (breastfeeding women interested in donating only): tick box (yes, no, unsure).

### 2.3 Procedure

Following ethical approval by Lifeblood (2024#18‐LNR) and The University of Queensland (2024/HE000885) Human Research Ethics Committees, eligible participants were recruited via paid Meta (Facebook, Instagram) ads. These targeted parents of young children living within 80 km of major cities in Australia (Brisbane, Sydney, Melbourne, Canberra, Adelaide, or Perth), where a Lifeblood or hospital milk bank collection point was operational (except Canberra). Purposive sampling was based on 2023 Australian registered births by state (ABS 2023). As fewer than 1% of women in Australia who give birth become milk donors (550 donors to Lifeblood in 2023), we aimed to recruit approximately 0.5% of each state's registered births.

Participant recruitment and survey completion occurred from March 2025 to April 2025. Before completing the survey, participants read information about the study and gave informed consent. The survey took approximately 5‐15 min to complete. Participants could opt‐in to a gift card draw at completion as a thank‐you for their participation. This incentive was included only in the Participant Information Sheet once the survey was accessed. Contact details for participants entering the draw were collected and stored separately to survey data to preserve participant anonymity.

### 2.4 Data Analysis

Responses were removed if they contained incomplete data (*n* = 37; demographic questions answered only), or respondents failed to meet eligibility criteria (*n* = 79 not pregnant or did not have an infant under 24 months; *n* = 38 donated milk to Lifeblood in previous 12 months; *n* = 3 lived outside target recruitment areas). One duplicate entry containing identical quantitative and free‐text responses to a previous entry was identified during content‐based data checks and removed.

Quantitative data were analysed using IBM SPSS (Version 30). Descriptive statistics were used to compute frequencies (*n*, %) overall and by State (given that different milk banks operate in each state, and access may also vary) for: general awareness of milk banks (all participants); awareness of local milk bank (all participants); preferred sources of information (pregnant women only); interest in donating and reasons for disinterest; feasibility of milk donation; and reasons for infeasibility for those women breastfeeding with infants only. Bivariate correlations using Spearman's Rho (ρ) examined associations between age (continuous) and categorical variables. To enable these analyses, categorical variables (see Appendix 1) were coded as follows:

- General awareness of milk banks: recoded from the original three‐point scale (yes, no, unsure) into a binary variable (1 yes, 2 No/Unsure). The original ‘unsure’ category was small (*n* = 47) and conceptually aligned with lack of awareness. This approach ensured sufficient cell sizes and simplified interpretation for correlational analysis.
- Level of interest: 1 Not interested, 2 Unsure, 3 Interested a little, 4 Interested a lot.
- Feasibility: 1 Very difficult, 2 Difficult, 3 Easy, 4 Very easy.

The resulting range for these variables was 1‐2 for awareness, where lower scores indicated greater general awareness of milk banks, and 1‐4 for interest and feasibility, where higher scores indicated greater interest and feasibility. Additional bivariate correlational analyses (Spearman's Rho) were undertaken between age and the four most frequently cited reasons for infeasibility, coded as 0 No (does not apply), 1 Yes (applies).

Chi‐square tests for independence were used for group comparisons that aligned with study aims, namely to explore whether general awareness of milk banks differed based on parental status (first‐time parents vs. not first‐time parents); blood donor status (blood/plasma donor vs. non‐donor); and if feasibility of donation differed based on parental status. Other variables were not included due to insufficient respondent numbers in each group (i.e. language, gender) or because they were not of interest to the milk bank operationally.

A significant result for analyses was determined by *p* < 0.05. Results of these comparisons are only reported in text if they were statistically significant. Chi‐square tests were chosen because both general awareness of milk banks and group variables (parental status and blood/plasma donor history) were categorical.

For free‐text narrative responses, we used a combination of reflexive and codebook thematic analysis (Braun and Clarke 2021) to inductively and deductively (based on the milk bank literature) generate codes and construct themes. One author reviewed all narrative free‐text responses and generated an initial coding framework to apply to the data. The resulting coding framework was reviewed, modified (e.g., remove duplication, clarify or refine coding), and agreed upon by two authors and added to qualitative data management software NVivo 13 (2020, R1). One author subsequently coded narrative free‐text data using this framework. The frequency with which themes occurred was quantified using content analysis.

**Figure 1:**
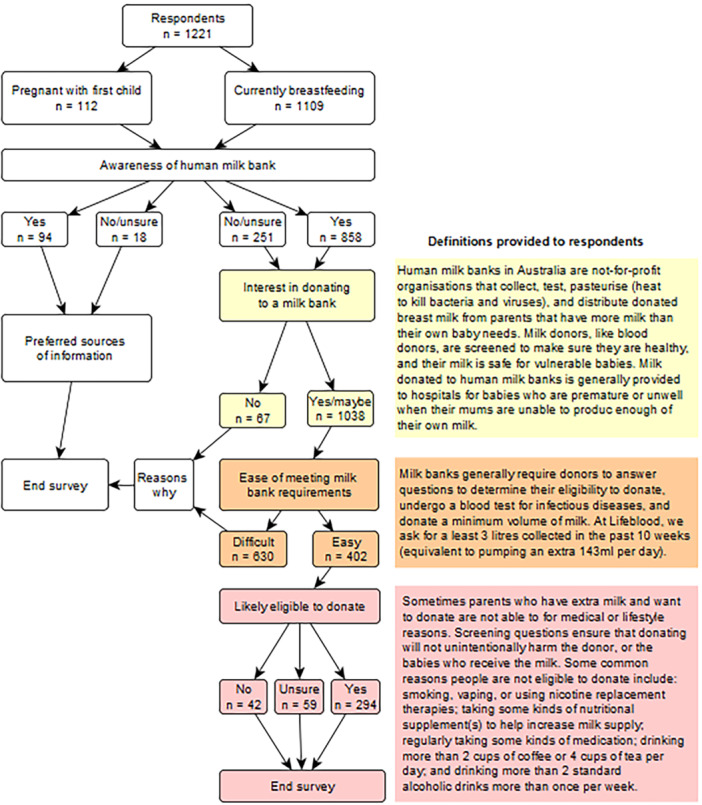
Summary of survey participation and definitions provided to participants.

## 3 Results

### 3.1 Participant Demographics

A total of 1221 survey responses were included in the analysis (Figure 1). Most participants resided in Victoria (*n* = 427, 35%) followed by New South Wales (*n* = 278, 22.8%) and Queensland (*n* = 224, 18.3%). The sample represented 0.3% to 1.3% of the registered births for each state in 2023. Table 1 shows demographic data for participants.

**Table 1:** Participant demographic data.

Descriptor	Respondents	
	*n*	%
Gender		
Woman/female	1214	99.4
Non‐binary or gender diverse	5	0.4
Undisclosed	2	0.2
State		
New South Wales	278	22.8
Australian Capital Territory	77	6.3
Victoria	427	35.0
Queensland	224	18.3
South Australia	69	5.7
Western Australia	132	10.8
Undisclosed	14	1.1
Primary language		
English	1207	98.9
Other^a^	14	1.1
First‐time parent		
Yes	606	49.6
No	604	49.5
Undisclosed	11	0.9
Age of youngest child (months)		
< 2	196	16.1
2–6	396	32.2
7–9	172	14.1
10–12	114	9.3
13–16	122	10.0
17–24	112	9.2
Not yet born	112	9.2
History of donating blood/plasma		
Yes	747	61.2
No	466	38.2
Undisclosed	8	0.7
Last blood/plasma donation (years)		
< 2	181	24.2
2–5	268	35.8
> 5	287	38.4
Undisclosed	11	1.4

^a^ Other languages spoken at home included Mandarin (*n* = 5),

Indonesian (*n* = 2), Cantonese (*n* = 1), Sinhalese (*n* = 1), French (*n* = 1),

German (*n* = 1), Swedish (*n* = 1) and Auslan (*n* = 1), all < 1%.

Mean age of participants was 33.7 years (SD = 4.04, range 19–48 years). Most identified as female (*n* = 1214, 99.4%) with English the primary language spoken at home (*n* = 1207, 98.9%). Half (*n* = 606, 49.6%) were first‐time parents. Most had an infant under 12 months of age (*n* = 878, 71.9%). Nine percent were pregnant with their first child (*n* = 112, 9.2%). Over half (*n* = 747, 61.2%) had donated blood or plasma, with most donating two or more years ago (*n* = 287, 73.4%).

### 3.2 Awareness of Milk Banks

#### 3.2.1 General Awareness of Milk Banks

Most participants (*n* = 953, 78%) had previously heard of a human milk bank (Table 2). This general awareness varied by State, ranging from 71.4% (Victoria) to 87% (South Australia). Fewer had heard of peer milk donation/milk sharing (*n* = 736, 60.3%) (Table 2).

**Table 2:** Awareness of milk donation.

Awareness	NSW	ACT	VIC	QLD	SA	WA	Total sample
	*n* = 278	*n* = 77	*n* = 427	*n* = 224	*n* = 69	*n* = 132	*n* = 1221
	n (%)	*n* (%)	*n* (%)	*n* (%)	*n* (%)	*n* (%)	*n* (%)
Heard of a HMB							
Yes	228 (82.0)	63 (81.8)	305 (71.4)	182 (81.3)	60 (87.0)	106 (80.3)	953 (78.0)
No	40 (14.4)	13 (16.9)	102 (23.9)	33 (14.7)	7 (10.1)	24 (18.2)	222 (18.2)
Unsure	10 (3.6)	1 (1.3)	20 (4.7)	9 (4.0)	2 (2.9)	2 (1.5)	47 (3.8)
Aware of local HMB							
Yes	19 (6.8)	5 (6.5)	40 (9.4)	30 (13.4)	11 (15.9)	27 (20.5)	135 (11.1)
No	50 (18.0)	33 (42.9)	65 (15.2)	34 (15.2)	10 (14.5)	18 (13.6)	210 (17.2)
Unsure	159 (57.2)	25 (32.5)	200 (46.8)	118 (52.7)	39 (56.5)	61 (46.2)	607 (49.7)
Tried to donate to local HMB							
Yes	4 (1.4)	1 (1.3)	12 (2.8)	9 (4.0)	5 (7.2)	12 (9.1)	45 (3.7)
No	12 (4.3)	4 (5.2)	26 (6.1)	17 (7.6)	5 (7.2)	12 (9.1)	77 (6.3)
Heard of peer milk donation							
Yes	166 (59.7)	56 (72.7)	260 (60.9)	135 (60.3)	43 (62.3)	71 (53.8)	736 (60.3)
No	97 (34.9)	17 (22.1)	156 (36.5)	81 (36.2)	23 (33.3)	60 (45.5)	441 (36.1)
Unsure	15 (5.4)	4 (5.2)	11 (2.6)	8 (3.6)	3 (4.3)	1 (0.8)	44 (3.6)

Abbreviations: ACT = Australian Capital Territory, HMB = Human Milk Bank, NSW = New South Wales, QLD = Queensland, SA = South Australia, VIC = Victoria, WA = Western Australia.

Participant age was negatively, but weakly, associated with general awareness of milk banks (*ρ* = −0.07, *n* = 1202, *p* < 0.03), with older participants more likely to have heard of a human milk bank.

#### 3.2.2 Awareness of Local Milk Banks

Most participants (*n* = 607, 49.7%) were unsure if a milk bank operated in their area. Only 11.1% (*n* = 135) were aware of a milk bank operating in their local area, with a range between States of 6.5% (Australian Capital Territory) to 20.5% (Western Australia; Table 2). Of those living in a postcode eligible to donate to Lifeblood (*n* = 998, 81.7%), 9% (*n* = 90) were aware of a milk bank operating in their area. Although 17.2% (*n* = 210) indicated that a milk bank did not operate in their area, postcodes provided (*n* = 193) suggest that 67 (34.7%) of these respondents resided in an area eligible to donate to Lifeblood.

Of participants aware of a local milk bank (*n* = 135), most identified one (*n* = 97, 71.8%) or two (*n* = 3, 2.2%) milk banks. The remainder believed a milk bank existed but could not recall its name (*n* = 29, 21.5%) or did not provide a response (*n* = 7, 5.2%). At the time of the survey, four milk banks were operational in the study areas (Lifeblood, PREM Milk Bank, Mercy Milk Bank, Mother's Milk Bank Charity), and one had previously operated (Queensland Milk Bank; 2012–2021). Of respondents who named a milk bank, 81 (60%) identified an operational service, six named a previously operating milk bank, and 13 responses could not be matched to a current or former milk bank.

#### 3.2.3 Previous Donation to a Milk Bank

Few participants (*n* = 45, 3.7%) had previously donated or attempted to donate (e.g., ineligible) to their local milk bank, with a range between States of 1.4% (New South Wales) to 9.1% (Western Australia; Table 2). Of participants who previously tried to donate milk, 33 (73.3%) had a blood/plasma donation history.

### 3.3 Preferred Sources of Information about Milk Donation

Participants who were pregnant with their first child (*n* = 112) were asked how likely they would be to obtain information about milk donation from various sources (Appendix 2, Figure 1). Most indicated their preferred source of information was Google (*n* = 76, 68.5%), followed by the Australian Breastfeeding Association website (*n* = 69, 61.6%) and healthcare professionals such as nurses, midwives, lactation consultants and/or general practitioners (*n* = 57, 50.9%). Social media (e.g., Facebook, Instagram; *n* = 36, 32.4%) and Lifeblood's website (*n* = 24, 21.8%) were least likely to be used as preferred sources of information about milk donation.

### 3.4 Interest in Milk Donation

Breastfeeding women (*n* = 1109) were provided with a definition of a milk bank (Figure 1) and asked questions related to their interest in donating milk (Table 4). Most (79.3%) indicated they were either a little (*n* = 498, 44.9%) or a lot (*n* = 381, 34.4%) interested in donating to a milk bank. Only 6% (*n* = 67) indicated they were not interested. The remainder (*n* = 159, 14.3%) were ‘unsure’ or did not provide a response (*n* = 4, 0.4%).

#### 3.4.1 Reasons for Not Being Interested in Milk Donation

Among the 67 participants not interested in donating milk, common reasons were having no excess milk to donate (*n* = 53, 79.1%), lack of time (*n* = 24, 35.8%), and planning to stop/already stopped pumping (*n* = 10, 14.9%). Religious or cultural concerns were not cited as a reason for participant's disinterest (Table 3, Appendix 2).

**Table 3:** Reasons for disinterest in or difficulty donating milk.

	*n*	%^a^
Reasons participants were ‘not at all’ interested in donating (*n* = 67)		
I don't have extra milk/more milk than my baby needs	53	79.1
It would be hard to find the time to donate	24	35.8
I have stopped pumping or plan to stop soon	10	14.9
I don't think I would be eligible to donate	7	10.4
I'm just not interested/It's not for me	7	10.4
Prefer to donate via peer milk donation of peer‐sharing groups	2	3
Religious or cultural reasons	0	0
Reasons participants believed donating would be ‘very difficult’ or ‘difficult’ (*n* = 630)		
I don't express enough extra milk to donate/express < 3 litres in 10 weeks	420	66.7
It would be hard to find time to express and/or donate	287	45.6
I use all the milk I express for my own baby (no extra milk)	191	30.2
I don't have space to store extra milk for donation	174	27.6
I don't think I would be eligible to donate milk	68	10.8
I don't want to do a blood test/I don't like needles	17	2.7
I don't feel confident answering screening questions in English	1	0.2
I don't want to answer screening questions	1	0.2

^a^ Participants could choose more than one response.

### 3.5 Feasibility of Donating Milk

Breastfeeding women who were interested, or may be interested, in donating milk (*n* = 1038) were given information about milk bank requirements (Figure 1) and asked how easy it would be for them to meet these requirements. Most (60.7%) indicated that meeting the stated milk bank requirements would be difficult (*n* = 493, 47.5%) or very difficult (*n* = 137, 13.2%). Donating was commonly perceived as difficult due to participants not having enough milk to donate (Table 3 and Appendix 2, Table 1). This included not having a minimum of 3 L over 10 weeks (*n* = 420, 66.7%) and not having any surplus to donate (*n* = 191, 30.2%). Other frequently cited reasons included not having enough time (*n* = 287, 45.6%) or storage space (*n* = 174, 27.6%). Approximately 11% believed they would be ineligible to donate (*n* = 68, 10.8%).

Associations between participant characteristics and the four most frequently cited reasons for donation being viewed as difficult were explored. Participant age was negatively, weakly associated with perceived ease of meeting milk bank requirements (*ρ* = −0.09, *n* = 1019, *p* < 0.003), with older participants more likely to experience difficulties. Age was also weakly associated with not having enough time (*ρ* = 0.11, *n* = 620, *p* < 0.006), with older participants less likely to have time to express and/or donate.

First‐time parents were more likely to identify not expressing a sufficient volume of milk for donation as a reason donating may be difficult for them, *x* ^2^ (1, *n* = 415) = 13.79, *p* < 0.001, *phi* = −0.11. Participants with multiple children were more likely to identify that it would be hard to find time to express and/or donate, *x* ^2^ (1, *n* = 284) = 13.99, *p* = 0.046, *phi* = 0.06.

Thirty‐nine percent (*n* = 402) of breastfeeding women interested in donating indicated that meeting milk bank requirements would be easy (*n* = 312, 30.1%) or very easy (*n* = 90, 8.7%). These participants were then shown a statement outlining eligibility requirements that commonly result in deferral (Figure 1) and asked whether they believed they would be eligible to donate. Most felt they would be eligible to donate (*n* = 294, 73.1%), representing 26.5% of the total sample (excluding respondents without infants). Some were unsure if they would be eligible (*n* = 59, 14.7%), and 10.4% (*n* = 42) believed they would be ineligible. Table 4 shows interest in donating, ease of meeting requirements and potential eligibility by State.

**Table 4:** Interest in donating by state.

Interest in donating		NSW	ACT	VIC	QLD	SA	WA	Total sample^a^
		*n* = 260	*n* = 69	*n* = 437	*n* = 388	*n* = 58	*n* = 119	*n* = 1109
		*n* (%)	*n* (%)	*n* (%)	*n* (%)	*n* (%)	*n* (%)	*n* (%)
How interested would you be in donating to a human milk bank	Not at all/unsure	58 (22.3)	6 (8.7)	82 (21.1)	39 (19.4)	10 (17.2)	28 (23.5)	226 (20.4)
	A little/a lot	201 (77.3)	62 (89.9)	304 (78.4)	162 (80.6)	48 (82.8)	91 (76.5)	879 (79.3)
How easy would it be for you to meet milk bank requirements	Very difficult/difficult	165 (63.5)	40 (58.0)	210 (54.1)	110 (54.7)	30 (51.7)	66 (55.5)	630 (56.8)
	Very easy/easy	77 (29.6)	27 (39.1)	150 (38.7)	79 (39.3)	26 (44.8)	40 (33.6)	402 (36.2)
Do you think you are eligible to donate	Yes	58 (22.3)	24 (34.8)	106 (27.3)	55 (27.4)	18 (31.0)	31 (26.1)	295 (26.5)
	No	6 (2.3)	1 (1.4)	16 (4.1)	10 (5.0)	4 (6.9)	5 (4.2)	42 (3.8)
	Unsure	11 (4.2)	2 (2.9)	25 (6.4)	13 (6.5)	4 (6.9)	3 (2.5)	59 (5.3)

Abbreviations: ACT = Australian Capital Territory, NSW = New South Wales, QLD = Queensland, SA = South Australia, VIC = Victoria, WA = Western Australia.

^a^ Excluding respondents without infants.

## 4 Discussion

This study surveyed pregnant women intending to breastfeed and women who were breastfeeding infants under 24 months to explore their awareness of milk banks, and for breastfeeding respondents, their interest in donating milk to a milk bank and their perceived feasibility of donating. General awareness of milk banks was high; however, specific awareness of local milk banks was low. While interest in milk donation was high, perceptions of feasibility varied and should be interpreted with caution due to an error in the survey item describing minimum daily donation volume (the total volume required—at least 3 L collected in 10 weeks—was reported correctly). Key reasons participants perceived donation as unfeasible comprised mostly less‐modifiable barriers (e.g., not having extra milk or time to express). Only approximately one‐quarter of participants believed they would be eligible to donate, yet this proportion far exceeds the number of donors currently to Australian milk banks, suggesting that efforts to raise awareness among mothers with surplus milk are warranted.

### 4.1 Awareness of Milk Banks and Milk Banking Services

While most (78%) had heard of milk banking prior to the survey, only 11% were aware of a milk bank operating in their area, and fewer than two‐thirds of those aware were able to name a currently operating milk bank. This limited awareness of local milk banks may reflect the relatively recent expansion of milk banking in Australia, as opportunities to donate milk before 2018 were limited (Australian Red Cross Lifeblood 2026). Alternatively, this finding aligns with the broader literature on potential donors, which shows a lack of awareness of the existence of milk banks (Mampane and Wolvaardt 2024; Smyk et al. 2021).

Regardless, this finding highlights the need to address this modifiable barrier by raising awareness amongst potential donors of local milk banks, potentially by leveraging the link that some participants made between milk and blood donation. Prior milk donation research also reflects this connection, showing high numbers of participants had a blood donor history (Hamilton and Middlestadt 2024; Osbaldiston and Mingle 2007), perceived similarities between the two donation processes (Mackenzie et al. 2013), and similar motives for blood and milk donors (Hyde et al. 2024). A US collaboration between a blood centre and milk bank showed that traditional and social media campaigns, along with promotional materials distributed to current and former donors, increased awareness (O'Rourke et al. 2021). Current/former blood or plasma donors are already familiar with donor screening processes, and if not eligible to donate milk themselves, may still promote it to others. For women deferred from blood donation during pregnancy and breastfeeding, milk donation may also help to maintain their donor identity and connection with the organisation (Thorpe et al. 2020).

### 4.2 Preferred Sources of Information About Milk Donation

Pregnant women were most likely to seek information about human milk donation online. This preference is consistent with Smyk et al. (2021), who reported that women would look to the internet to expand their knowledge of milk donation. This may also reflect systematic review findings that online information is preferred, given inadequate resources and lack of promotion of donation in health care settings (Li et al. 2024).

Approximately 50% of pregnant women indicated they would seek information from health professionals about milk donation. This aligns with prior research showing women want information and support from health professionals about milk banking/donation (Bhoola and Biggs 2021; Hamilton and Middlestadt 2024; Mackenzie et al. 2013; Virano et al. 2017; Zhang et al. 2024). Studies in Brazil show that health professionals shape women's views about donation, raise awareness early (e.g., during routine pregnancy care), and inform mothers with oversupply about local milk banks and the donation process (Meneses et al. 2017; Pimenteira Thomaz et al. 2008). Providing resources and education for health professionals unfamiliar with milk bank processes may increase their confidence and likelihood they may identify potential donors (Kundisova et al. 2019; Pimenteira Thomaz et al. 2008).

### 4.3 Interest in and Feasibility of Milk Donation

Approximately three‐quarters of breastfeeding women in this study were interested in donating to a milk bank. While this may reflect the self‐selected nature of our sample, these rates are higher than rates of willingness to donate to a milk bank reported in studies of lactating mothers in South Africa and India (~ 50%; Bhoola and Biggs 2021; Jayakrishna et al. 2024), but consistent with studies of lactating mothers in China (76.7%; Tian et al. 2021), and mothers of infants < 24 months in Vietnam (75.7%; Nguyen et al. 2025).

Despite their interest, only a quarter of these women thought donating would be feasible—that is, they perceived meeting milk bank requirements as easy and believed they would be eligible to donate based on criteria given. Most commonly, not having extra milk or time to donate impacted potential donors' interest and perceived feasibility of donating milk. Both of these less‐modifiable barriers feature prominently in prior research amongst potential milk donors (e.g., Bhoola and Biggs 2021; Brown et al. 2025; Hamilton and Middlestadt 2024; Mackenzie et al. 2013; Tian et al. 2021; Virano et al. 2017).

Notably, that potential donors can have a limited amount of milk to give regardless of their interest or motivation to donate is a unique barrier compared to blood or other donation types. Blood donation is designed not to negatively impact the donor's health, and the volume donated is regenerated without conscious effort by the donor. Milk for donation must be intentionally expressed and stored. Whilst some women produce more milk than their own baby needs naturally or by circumstance (e.g. after preterm birth when infant's intake is limited), milk production is generally physiologically regulated to match the baby's needs. Oversupply can increase the risk of mastitis (Cullinane et al. 2015), so it is justifiable that some respondents expressed concern for potential risks of increasing milk supply solely for the purpose of donation (Appendix 2, Table 1). Milk banks must balance encouraging donation to meet demand whilst ensuring that donors do not give milk at the expense of their own or their baby's health.

Other potential donors, despite being interested in donating, indicated that finding time to donate would be too difficult. While we did not further explore what potential donors meant by not having time, prior qualitative work with potential milk donors suggests that difficulty or complexity of the process, effort required, time needed, mental capacity, and personal resources may all be considered in an individual's assessment of whether they have time and capability to donate (Newman et al. 2025). As with blood/plasma donation, to reduce perceived barriers for potential donors, such as lack of time or too much effort required, it is important to emphasise in communications with potential donors the aspects of the donation process that are easy or convenient (Hyde et al. 2024). For example, in Lifeblood's milk service, initial eligibility screening of potential donors is via a brief online survey and then a telephone interview. For convenience, blood testing, further screening, and collection of milk occur through an in‐home visit from a donor coordinator. For potential donors who have concerns about the process or their capability of donating, it may be useful to offer a realistic preview of the process online and include stories and tips from current donors describing how they navigate potential challenges.

An additional, less‐modifiable barrier that made donation infeasible for a third of potential donors was not having space to store extra milk for donation. While limited freezer space is a driver for some to donate to a milk bank or via peer‐sharing (Newman et al. 2026; Perrin et al. 2016), or purchase/borrow additional freezer space (Hyde et al. 2023; Perrin et al. 2016), others may not have the resources to overcome this barrier. A lack of freezer space has been identified as preventing milk donation in prior research among donors and potential donors (Brown et al. 2024, 2025; Gutierrez dos Santos et al. 2024; Newman et al. 2025). For potential donors who have concerns about a lack of space to store extra milk, milk banks could consider increasing access to collection points or offering greater frequency of collection.

While these less‐modifiable barriers may be insurmountable for many otherwise interested, our findings suggest there are many more women than those who currently donate who may be able to become donors. Approximately 300,000 women give birth in Australia each year (ABS 2025), with approximately 500 of these women donating to Lifeblood's milk bank annually (internal data), representing 0.16% of women giving birth annually. The finding that a quarter of women in this study thought donation was feasible is therefore useful from a forecasting perspective.

### 4.4 Strengths and Limitations

Findings should be interpreted with limitations in mind. Participants self‐selected into the study and were likely more motivated to engage with milk donation compared to non‐participants, although their average age was similar to mothers who gave birth in Australia (31.2 years; AIHW 2025) and to Lifeblood milk donors (32.4 years; Klein et al. 2025). Less than 2% of participants primarily spoke a non‐English language at home, indicating limited cultural diversity. A high proportion of former blood/plasma donors participated, and findings may therefore reflect the awareness and barriers of those more familiar with Lifeblood and donation than individuals without prior donor experience.

Although the survey used in this study was informed by qualitative data from potential donors, it was not reviewed with the target population for face validity. A typographical error in the survey may have influenced participants' perceptions of feasibility. While the total minimum donation requirement of 3 L over 10 weeks was stated correctly, the parenthetical illustration of the equivalent daily volume incorrectly reported this as 143 mL per day instead of 43 mL per day. Online recruitment and surveys increased accessibility and geographic reach but may have excluded women who do not use social media. State‐based differences may also reflect eligibility bias, as some eligible women may already be donors and therefore were not represented.

Although the coding structure for free‐text narrative responses was agreed upon by two authors, only one author coded responses. Because our focus was on understanding awareness, interest, and feasibility of potential donors, other relevant factors potentially impacting donation were unexplored. Specifically, cultural, linguistic, or socioeconomic factors; detailed knowledge about milk banking and processes; motives for donation; specific cultural or religious beliefs (not cited by participants in this study as reasons for disinterest in donating to a milk bank); and perspectives of health professionals, were unexplored. These factors warrant in‐depth examination in future research.

## 5 Conclusion

In conclusion, this is the first Australian study on potential donors at a time when milk banks are operational in most of the areas surveyed. It shows a lack of awareness about local milk banks in Australia, and that preferred sources of information about milk banking include online resources and health professionals. Despite a high level of interest in donating to a milk bank, perceptions of the feasibility of donating varied, with women being primarily concerned that they would not have enough milk to donate, time, or storage space. To ensure the sustainability of milk banks in Australia and elsewhere, awareness raising of local milk banks, promotion of donation information amongst healthcare professionals, and modifications to improve the feasibility of donation need to occur if the number of potential donors is to be increased.

## Author Contributions

All authors were involved in project design. L.D.K., B.M.M., and V.C. obtained funding for the project. L.D.K. and B.M.M. were responsible for project management. C.N. and A.E. were responsible for protocol development. C.N. and L.D.K. were responsible for data collection. C.N., M.K.H., and A.E. were responsible for data analysis. All authors were involved in draft manuscript writing and read and approved the final manuscript.

## Conflicts of Interest

MKH, CN, AE, VC, BMM, and LDK are current or former employees or affiliates of Australian Red Cross Lifeblood, which provides pasteurised donor human milk to hospitals in Australia. The authors do not have any other conflicts of interest to declare.

## Supporting information

Supporting File

## Data Availability

The data that support the findings of this study are available on request from the corresponding author. The data are not publicly available due to privacy or ethical restrictions. Data supporting the results of this study are available on request from the research team pending approval from the Lifeblood Human Research Ethics Committee. Data are not publicly available due to privacy or ethical restrictions.
