# Supplementary material for: Potential Donors' Awareness and Perceived Feasibility of Donating Human Milk in Australia
Source: Matern Child Nutr. 2026 Jul 1;22(3):e70224. doi: 10.1111/mcn.70224 (PMC13320368; doi:10.1111/mcn.70224)
Supplement: Supplementary file 1 — Supporting File [file MCN-22-e70224-s001.docx]

Appendix 1

**Potential donor survey**

| **Section Header** | **Variable / Field Name** | **Field Label** | **Choices, Calculations, OR Slider Labels** | **Branching Logic (Show field only if...)** |
| --- | --- | --- | --- | --- |
| Eligibility check | elig1 | Which statement best applies to you? | 1. I am pregnant 2. I am breastfeeding/expressing milk for a child under 24 months of age 3. I am pregnant and breastfeeding/expressing milk for a child under 24 months of age 4. None of the statements apply to me | 4 go to ex1  1 to 3 got to elig2 |
|  | elig2 | Have you donated milk to Lifeblood in the last 12 months? | 1. Yes 2. No | 1 go to ex2  2 go to ‘About you’ section |
|  | ex1 | Thank you for your interest in the survey. Unfortunately, you do not meet the criteria to participate.  Information about milk donation can be found here: [link to Lifeblood milk bank page] |  |  |
|  | ex2 | Thank you for your interest in the survey. Unfortunately, you do not meet the criteria to participate.  If you have donated milk to Lifeblood in the last 12 months, we invite you to complete our survey for milk donors [link to current donor survey] |  |  |
| About you | postcode_v1 | What is your home postcode? | Free-text (limit to 4 characters) |  |
|  | language_v1 | What is your primary language spoken at home? | 1. English 2. Other | 1 go to age_v1  2 got to language_other_v1 |
|  | language_other_v1 | Please state primary language spoken at home | Free-text |  |
|  | age_v1 | What is your age? | Free-text (limit to 2 characters) |  |
|  | gender1_v1 | With which gender do you most identify? | 1. Woman or Female 2. Man or male 3. Non-binary or Gender diverse 4. Other 5. Prefer not to disclose | 1 to 3, 5, go to firsttimeparent_v1  4 go to gnder_other_v1 |
|  | gnder_other_v1 | If other, please state: | Free-text |  |
|  | blooddonor_v1 | Have you previously donated blood or plasma at Australian Red Cross Lifeblood? | 1. Yes 2. No | 1 go to blooddonor_when_v1  2 go to firsttimeparent_v1 |
|  | blooddonor_when_v1 | When was your last blood or plasma donation? | 1. In the last two years 2. 2 - 5 years ago 3. More than 5 years ago |  |
|  | firsttimeparent_v1 | Are you a first-time parent? | 1. Yes 2. No |  |
|  | childage | How old is your youngest child? | 1. less than 2 months old 2. 2 - 6 months old 3. 7 - 9 months old 4. 10- 12 months old 5. 13 - 18 months old 6. 17 - 24 months old 7. my child is not yet born | 1 to 6 go to Green section  7 go to Blue section |
| Blue section = If pregnant only |  |  |  |  |
| What do you know about milk donation? | preg_aware | Before seeing the advertisement for this survey, had you heard of a human milk bank? | 1. Yes 2. No 3. I'm not sure | 1 go to preg_qual_aware  2 to 3 go to aware_milksharing |
|  | preg_aware1 | Do you know if there is a human milk bank in your area? | 1. Yes, I know there is a human milk bank in my area 2. No, there is not a human milk bank in my area 3. I'm not sure if there is a human milk bank in my area | 1 go to preg_aware2  2 to 3 go to aware_milksharing |
|  | preg_aware2 | What's the name of your local milk bank? | Free-text |  |
|  | aware_milksharing | Have you heard of peer milk donation or milk-sharing (i.e. giving breast milk directly to another family) through online groups such as HumanMilk4HumanBabies or Eats on Feets? | 1. Yes 2. No 3. I’m not sure |  |
| If you wanted to find more information about milk donation, how likely would you go to the following sources? | preg_info1 | Healthcare professional (i.e. GP, Midwife, Lactation Consultant, Child and Family Health Nurse) | 1. Not at all 2. Maybe 3. Definitely |  |
|  | preg_info2 | Google | 1. Not at all 2. Maybe 3. Definitely |  |
|  | preg_info3 | Social media (i.e. Facebook, Instagram, TikTok) | 1. Not at all 2. Maybe 3. Definitely |  |
|  | preg_info4 | Lifeblood website | 1. Not at all 2. Maybe 3. Definitely |  |
|  | preg_info5 | Australian Breastfeeding Association website | 1. Not at all 2. Maybe 3. Definitely |  |
|  | preg_info6 | Other | 1. Not at all 2. Maybe 3. Definitely | 1 go to Thank you section  2 to 3 go to preg_info_other |
|  | preg_info_other | Please state other source of information you would use to find information about milk donation | Free-text |  |
| Green section = If have infant only |  |  |  |  |
| What do you know about milk donation? | aware | Before seeing the advertisement for this survey, had you heard of a human milk bank? | 1. Yes 2. No 3. I'm not sure | 1 go to qual_aware  2 to 3 go to aware_milksharing |
|  | aware1 | Do you know if there is a human milk bank in your area? | 1. Yes I know there is a human milk bank in my area 2. No, I know there is not a human milk bank in my area 3. I don't know if there is a human milk bank in my area | 1 go to aware2  2 or 3 go to aware_milksharing |
|  | aware2 | What's the name of your local milk bank? | Free-text |  |
|  | aware3 | Have you donated or tried to donate to this or another milk bank? | 1. Yes 2. No |  |
|  | aware_milksharing | Have you heard of peer milk donation or milk-sharing (i.e. giving breast milk directly to another family) through online groups such as HumanMilk4HumanBabies or Eats on Feets? | 1. Yes 2. No 3. I'm not sure |  |
| Human milk banks in Australia are not-for-profit organisations that collect, test, pasteurise (heat to kill bacteria and viruses), and distribute donated breast milk from parents that have more milk than their own baby needs. Milk donors, like blood donors, are screened to make sure they are healthy, and their milk is safe for vulnerable babies. Milk donated to human milk banks is generally provided to hospitals for babies who are premature or unwell when their mums are unable to produce enough of their own milk. | interest | How interested would you be in donating your breast milk to a human milk bank? | 1. Not at all 2. Unsure 3. A little 4. A lot | 1 go to interest_no  2 to 4 go to interest_ease |
|  | interest_no | Please indicate your reasons for not being interested (check all that apply) | 1. I don't have extra milk / more milk than my baby needs 2. I don't think I would be eligible to donate 3. It would be hard to find time to donate 4. I have stopped pumping or plan to stop pumping soon 5. Religious or cultural reasons 6. Prefer to donate via peer milk donation or peer-sharing groups 7. I'm just not interested / It's not for me 8. Other | 1 to 7 go to Thank you section  8 go to interest_no_other |
|  | interest_no_other | Please state 'other' reasons why you would not be interested | Free-text | Go to Thank you section |
| Milk banks generally require donors to answer questions to determine their eligibility to donate, undergo a blood test for infectious diseases, and donate a minimum volume of milk. At Lifeblood, we ask for at least 3 litres collected in the past 10 weeks (equivalent to pumping an extra 143 mL per day). **Please note that 143 mL per day (rather than 43 mL per day) is a typographical error in the survey.* | interest_ease | How easy do you think this would be for you to do? | 1. Very difficult 2. Difficult 3. Easy 4. Very easy | 1 or 2 go to interest_difficult  3 or 4 go to eligible1 |
|  | interest_difficult | Why do you think this would be difficult? (check all that apply) | 1. I use all the milk I express for my own baby (no extra milk) 2. I don't express enough extra milk to donate / express < 3 litres in 10 weeks 3. I don't have space to store extra milk for donation 4. I don't think I would be eligible to donate milk 5. I don't want to do a blood test / I don't like needles 6. It would be hard to find time to express and/or donate 7. I don't feel confident answering screening questions in English 8. I don't want to answer screening questions 9. Other | 1 to 8 go to Thank you section  9 go to interest_difficult_other |
|  | interest_difficult_other | Please state the 'other' reasons why it would be difficult | Free-text |  |
| Sometimes parents who have extra milk and want to donate are not able to for medical or lifestyle reasons. Screening questions ensure that donating will not unintentionally harm the donor, or the babies who receive the milk. Some common reasons people are not eligible to donate include:  - Smoking, vaping, or using nicotine replacement therapies - Taking some kinds of nutritional supplement(s) to help increase your milk supply  - Regularly taking some kinds of medication - Drinking more than 2 cups of coffee or 4 cups of tea per day - Drinking more than 2 standard alcoholic drinks more than once per week | eligible1 | Based on this information, do you think you are eligible to donate milk? | 1. Yes 2. No 3. I’m not sure |  |
| To thank you for your time to complete the survey, we invite you to enter a draw to win 1 of 10 gift vouchers valued at $100. Winners of the draw will be notifed via email in May 2025. Entry into the draw is optional. If you decide to enter the draw, your contact details will not be linked to your survey responses. This ensures your survey responses remain anonymous. | draw1 | Would you like to enter the draw? | 1. Yes 2. No | 1 go to draw2  2 thank you and exit |
|  | draw2 | Please provide your first name | Free-text |  |
|  | draw3 | Please provide your email address | Free-text | Thank you and exit |

Appendix 2

**Table 1:** Qualitative responses

| **Reasons for not being interested in donating (n = 9)** | ***n*** |
| --- | --- |
| Disliked expressing milk | 2 |
| Did not express milk | 1 |
| No excess milk to donate | 1 |
| Prescribed mediation made them ineligible | 1 |
| Rules were too complicated | 1 |
| No time to drive fresh milk to the milk bank | 1 |
| Exclusively feeding another baby via informal milk sharing | 1 |
| Breast milk should not be given away without compensation | 1 |
| **Reasons why donating may be difficult (n = 74)** | ***n*** |
| Don't express | 14 |
| Concerned about impact of inducing oversupply | 10 |
| No access to milk bank | 9 |
| Too much effort involved | 8 |
| Dislike/difficulty using breast pump | 5 |
| Unable to drop off milk/travel | 5 |
| No excess milk to donate | 4 |
| Three litres in 10 weeks too much | 3 |
| Not eligible (due to caffeine/alcohol intake or medication) | 3 |
| Easier/prefer to donate informally via Facebook | 3 |
| Maybe when baby is older | 3 |
| Want more information | 2 |
| Need better breast pump and more storage space | 2 |
| Almost finished breastfeeding | 1 |
| Lack of time - returning to work | 1 |
| Don't want to restrict diet | 1 |
| Baby too old (>12 months) | 1 |
| Concern milk not suitable (high lipase) | 1 |
| Want stash stored for my baby | 1 |

**Figure 1:** Percentage of respondents (without infants) indicating preferred sources of information


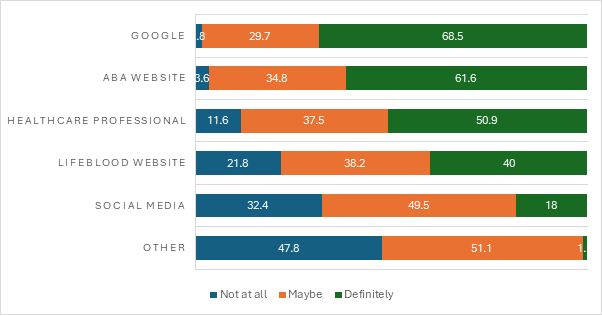
Note: An example of an ‘other’ response is “friends who have donated or received donations.”
